# The Influence of Climate on Pulmonary Phthisis

**Published:** 1895-11

**Authors:** W. H. Phillips

**Affiliations:** Thomasville, Ga.


### THE INFLUENCE OF CLIMATE ON PULMONARY PHTHISIS.

W. H. Phillips, M. D., Thomasville, Ga.

In so short a paper I do not care to take up the theory and diagnosis of pulmonary tuberculosis, the literature on those topics being extensive, and all are familiar with the important points.

Does climate have any effect on phthisis? Yes, if the patient is given the opportunity to avail himself of its benefits, and the disease has not developed beyond a stage where an arrest in its progress is impossible.

"Climate seems to exert an influence for or against the development of pulmonary phthisis. The prevalence of this disease is less in climates either uniformly warm and dry or uniformly cold and dry than in those which are moist and subject to frequent alternations of cold and warmth."

When are the best results obtainable from a change of climate in phthisis? In the early stages, before cavities have developed. It is folly to send patients in the last stages of the disease away in the hope of bringing about a change in their condition; the fatigue of the journey and inconveniences that such patients are compelled to submit to in traveling are such that more harm results than would have been the case had the patient remained at home.

When there is evidence of pulmonary invasion, such as slight cough of a "hacking" character, and which may be of a single effort aggravated by change of posture or from deep breathing, usually unaccompanied by expectoration and heard infrequently during the twenty-four hours. As the cough increases, there is more or less expectoration of a mucous nature, following this there may be some febrile disturbance, and the cough becomes more frequent and pronounced; emaciation, loss of appetite, sweat, and a train of other symptoms.

Don't wait until the bacillius is present in the expectoration, but after a careful examination of the patient inform him of his condition and the results to be obtained, that a cure is possible by residing in a locality suited to his or her case, at least a portion of the year, allowing patient to come North during the hot summer months. Experience proves that a majority of these cases do better in a region lying between a cold and warm climate, having favorable elements aside from temperature. The chief points are that it is not liable to either extreme of temperature; that there is not danger of exposure to cold winds, and that it is not liable to a continuance of unfavorable weather. There should be an agreeable temperature, dryness of atmosphere and a healthy soil well adapted to dryness.

Of the many places recommended perhaps southwestern Georgia offers more advantages than others. That portion of the State has a sufficient elevation to give a good natural drainage; is located well inland, thus being free from sudden changes or fogs which prevail on the coast during the winter months; is surrounded by thick pine forests, which must be considered of value.

Thomasville, of all other places, has received more patronage from the profession because of its climate, location, and accommodations.

It has a remarkably equable temperature and dry atmosphere. During the winter months the daily range is from 40 to 70 degrees, with cool nights, and periods of from twenty to twenty-five days without rain.

There is a sandy, porous soil, dampness rapidly disappearing, in fact, after a heavy rain, water is seldom seen standing in pools. The days are bright and sunshiny, with balmy breezes from the south (the prevailing winds are from that direction), so that the most delicate of invalids can practically live out-of-doors.

There are a number of parks in the pines where patients may walk, drive, or rest, and enjoy the sun and soft breezes, filled with the odor of the pine tree.

Perhaps the best natural roads in the South are to be found here; they are smooth, without stone or pebble, inviting riding, driving, and bicycling, through the groves of pine.

There is a beautiful boulevard fourteen miles in length, which completely encircles the city at about two miles' distance.

A gentleman who has traveled extensively, on being asked his opinion of the roads said: They are the best natural roads he ever saw, and as an evidence of how much he appreciated the privilege of driving through the pines I give the following memoranda made by him.

1889. Stayed 107 days, minus 15 Sundays, leaves 92 driving days; drove 86 days.

1891. Stayed 73 days, minus 10 Sundays, leaves 63 driving days; drove 54 days.

1892. Stayed 80 days, minus 11 Sundays, leaves 69 driving days; drove 59 days.

1893. Stayed 91 days, minus 13 Sundays, leaves 78 days, and spent three days in Florida; out of the 75 remaining days, drove and rode bicycle 68 days.

The drives average fourteen miles and bicycle rides twenty miles. The same gentleman has spent five winters in Florida, two in California, one in Mexico, one in Cuba, one along the

Riviera, in Algiers, and four seasons in Thomasville. He also said: "I do not hesitate to say that Thomasville in many respects is the most delightful winter place I have ever visited. The drives, the superb hotels, the balmy air, the hospitality of its citizens make it one of the most charming resorts."

